# Preclinical Characterization of DPI-4452: A ^68^Ga/^177^Lu Theranostic Ligand for Carbonic Anhydrase IX

**DOI:** 10.2967/jnumed.123.266309

**Published:** 2024-05

**Authors:** Frédéric Massière, Norbert Wiedemann, Inês Borrego, Aileen Hoehne, Frank Osterkamp, Matthias Paschke, Dirk Zboralski, Anne Schumann, Anne Bredenbeck, Franck Brichory, Antoine Attinger

**Affiliations:** 1 Debiopharm International SA, Lausanne, Switzerland; and; 2 3B Pharmaceuticals GmbH, Berlin, Germany

**Keywords:** CAIX, ^177^Lu, ^68^Ga, dog, mouse

## Abstract

The membrane protein carbonic anhydrase IX (CAIX) is highly expressed in many hypoxic or von Hippel-Lindau tumor suppressor–mutated tumor types. Its restricted expression in healthy tissues makes CAIX an attractive diagnostic and therapeutic target. DPI-4452 is a CAIX-targeting cyclic peptide with a DOTA cage, allowing radionuclide chelation for theranostic purposes. Here, we report CAIX expression in multiple tumor types and provide in vitro and in vivo evaluations of ^68^Ga-labeled DPI-4452 ([^68^Ga]Ga-DPI-4452) and ^177^Lu-labeled DPI-4452 ([^177^Lu]Lu-DPI-4452). **Methods:** CAIX expression was assessed by immunohistochemistry with a panel of tumor and healthy tissues. The molecular interactions of complexed and uncomplexed DPI-4452 with CAIX were assessed by surface plasmon resonance and cell-binding assays. In vivo characterization of radiolabeled and nonradiolabeled DPI-4452 was performed in HT-29 colorectal cancer (CRC) and SK-RC-52 clear cell renal cell carcinoma (ccRCC) human xenograft mouse models and in healthy beagle dogs. **Results:** Overexpression of CAIX was shown in several tumor types, including ccRCC, CRC, and pancreatic ductal adenocarcinoma. DPI-4452 specifically and selectively bound CAIX with subnanomolar affinity. In cell-binding assays, DPI-4452 displayed comparably high affinities for human and canine CAIX but a much lower affinity for murine CAIX, demonstrating that the dog is a relevant species for biodistribution studies. DPI-4452 was rapidly eliminated from the systemic circulation of beagle dogs. The highest uptake of [^68^Ga]Ga-DPI-4452 and [^177^Lu]Lu-DPI-4452 was observed in the small intestine and stomach, 2 organs known to express CAIX. Uptake in other organs (e.g., kidneys) was remarkably low. In HT-29 and SK-RC-52 xenograft mouse models, both [^68^Ga]Ga-DPI-4452 and [^177^Lu]Lu-DPI-4452 showed tumor-selective uptake; in addition, [^177^Lu]Lu-DPI-4452 significantly reduced tumor growth. These results demonstrated the theranostic potential of DPI-4452. **Conclusion:** DPI-4452 selectively targets CAIX. [^68^Ga]Ga-DPI-4452 and [^177^Lu]Lu-DPI-4452 localized to tumors and were well tolerated in mice. [^177^Lu]Lu-DPI-4452 demonstrated strong tumor growth inhibition in 2 xenograft mouse models. Thus, the 2 agents potentially provide a theranostic approach for selecting and treating patients with CAIX-expressing tumors such as ccRCC, CRC, and pancreatic ductal adenocarcinoma.

## Visual Abstract

**Figure:**
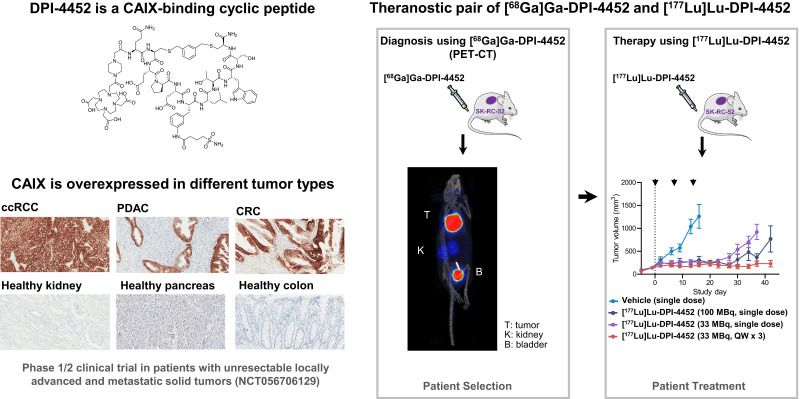

Carbonic anhydrases (CAs) catalyze the reversible hydration of carbon dioxide to bicarbonate ions and protons. Of the 12 catalytically active CAs in humans, 4 are located on the extracellular surface. Two of these, CAIX and CAXII, are highly expressed in tumors (1,2) and contribute to maintenance of the acidic tumor microenvironment (3). Many healthy tissues express CAXII, whereas normal CAIX expression is restricted to the gastrointestinal epithelia (1,2,4–6).

In clear cell renal cell carcinomas (ccRCC), impaired von Hippel-Lindau tumor suppressor function causes deregulation of hypoxia-inducible factor 1α expression, resulting in constitutive CAIX expression (7). Transcriptional regulation by hypoxia-inducible factor 1α (8) leads to CAIX overexpression in hypoxic conditions (2) and aberrant expression in some solid tumors, including colorectal cancer (CRC), breast cancer, and pancreatic ductal adenocarcinoma (PDAC) (2,9–11). CAIX overexpression is associated with tumor progression, poor prognosis, and metastasis development (9,12). With restricted expression in healthy tissues and near ubiquitous cell surface expression in ccRCC and hypoxic tumors (7), CAIX represents a promising diagnostic and therapeutic target (7,13).

DPI-4452 is a CAIX-targeting peptidomimetic carrying a DOTA cage (Supplemental Fig. 1; supplemental materials are available at http://jnm.snmjournals.org), allowing chelation with radionuclides such as ^68^Ga ([^68^Ga]Ga) or ^177^Lu ([^177^Lu]Lu) for theranostic purposes. Here, we report on the tumor-specific expression of CAIX and preclinical characterization of DPI-4452 in proof-of-concept studies, including safety, biodistribution, pharmacokinetics, and antitumor efficacy.

## MATERIALS AND METHODS

Detailed information on materials and methods are available in the Supplemental Materials and Methods.

### Immunohistochemistry

Staining of human healthy and tumor tissue microarrays (US Biomax Inc.) using an anti-CAIX antibody (clone M75; Creative Biolabs) was performed. Scoring by pathologists followed the methodology of Ilie et al. (14).

### Surface Plasmon Resonance

Recombinant CA proteins (Sino Biologic) were immobilized on Biacore CM5 sensor chips or biotin CAPture chips (Biacore). DPI-4452 binding was measured using the Biacore Liquid System (Biacore), and binding kinetic parameters were calculated.

### Cell-Binding Assays

Chinese hamster ovary cells (InSCREENex GmbH) expressing human CAIX (hCAIX), canine CAIX (cCAIX), or murine CAIX (mCAIX) were generated and incubated in the presence of ^111^In-labeled DPI-4452 ([^111^In]In-DPI-4452) for 8 h. Binding was assessed using a γ-counter and normalized to protein concentration.

### Animal Models

All animal experiments were performed by Minerva Imaging under a license approved by the National Animal Inspectorate under the Ministry of Environment and Food of Denmark and in accordance with ARRIVE guidelines (15).

For xenograft models, female Swiss nude mice were subcutaneously injected with between 2 × 10^6^ and 5 × 10^6^ human HT-29 CRC cells (HTB-28; American Type Culture Collection) or SK-RC-52 ccRCC cells (Memorial Sloan Kettering Cancer Center) (16). Mice were weighed and tumors were measured 2 or 3 times per week. Healthy beagle dogs and xenograft-bearing mice were injected intravenously with radiolabeled DPI-4452. Blood radioactivity counts or DPI-4452 plasma concentration data determined by liquid chromatography–tandem mass spectrometry were used for pharmacokinetic analyses. Biodistribution was assessed using PET/CT and SPECT/CT.

### Statistical Analyses

Statistical analyses were performed using GraphPad Prism software.

## RESULTS

### CAIX Is Overexpressed in Multiple Human Tumor Types

The suitability of CAIX as a tumor biomarker and therapeutic target was investigated using immunostaining of human tissue microarrays. Healthy stomach and pericardial mesothelial tissues displayed the highest CAIX expression, with more modest staining in the skin, tumor-adjacent normal ovary tissue, and small intestine. High CAIX expression was observed in samples of ccRCC, CRC, squamous non–small cell lung cancer, and PDAC, with no or low expression in the respective normal tissues. Samples of squamous cell carcinomas of the head and neck and triple-negative breast cancer also had high CAIX expression (Table 1; Supplemental Fig. 2).

**TABLE 1.** CAIX Expression Measured by Immunohistochemistry in Multiple Tumor and Control Tissues

Tissue	H score*	ccRCC^†^	CRC^†^	Sq. NSCLC^†^	PDAC^†^	SCCHN^†^	TNBC^†^
Malignant	>150	83% (25/30)	29% (25/85)	19% (15/79)	40% (26/65)	23% (14/60)	19% (10/54)
	>100	83% (25/30)	41% (35/85)	20% (16/79)	51% (33/65)	33% (20/60)	24% (13/54)
	>40*	87% (26/30)	52% (44/85)	42% (33/79)	60% (39/65)	62% (37/60)	37% (20/54)
Healthy control	>150	0% (0/30)	0% (0/21)	0% (0/22)	0% (0/4)	ND	ND
	>100	0% (0/30)	0% (0/21)	0% (0/22)	0% (0/4)	ND	ND
	>40	0% (0/30)	0% (0/21)	0% (0/22)	0% (0/4)	ND	ND

* H score value used as described by Ilie et al. (14). Number (and calculated percentage) of cases of given H score category also includes positive cases from all categories with higher H scores.

† Data are reported as percentages, with number of positive samples/total number of samples assessed in parentheses.

Sq. NSCLC = squamous non–small cell lung cancer; PDAC = pancreatic ductal adenocarcinoma; SCCHN = squamous cell carcinoma of head and neck; TNBC = triple-negative breast cancer; ND = not determined.

### DPI-4452 Binds Potently and Specifically to CAIX with Minimal Internalization

DPI-4452 inhibited recombinant hCAIX enzymatic activity in in vitro colorimetric assays (inhibitory concentration of 50%, 130 nM; 95% CI, 90—190 nM) (Supplemental Fig. 3). In surface plasmon resonance experiments, DPI-4452—with or without chelating lutetium or gallium ions—showed a high affinity for hCAIX, with a subnanomolar dissociation constant (K_D_) and a dissociation half-life of 99–123 min (Table 2). [^nat^Lu]Lu-DPI-4452 did not bind other extracellular CA proteins (Table 3). Furthermore, DPI-4452 showed no interaction with an in vitro off-target receptor panel of 55 targets (Supplemental Fig. 4; Supplemental Table 1). Thus, DPI-4452 is considered both highly selective and specific for CAIX. Incubation of a fluorophore-labeled DPI-4452 derivative with CAIX-expressing HT-29 cells revealed cell surface binding and minimal internalization over 72 h (Supplemental Figs. 5 and 6), consistent with other CAIX-targeting agents (17).

**TABLE 2.** Affinity of DPI-4452 for hCAIX

Compound	K_D_ (nM)	Dissociation half-life (min)
DPI-4452	0.25	99
[^nat^Lu]Lu-DPI-4452	0.16	123
[^nat^Ga]Ga-DPI-4452	0.2	112

Affinities and dissociation kinetics were measured by surface plasmon resonance assay.

**TABLE 3.** Affinity of [^nat^Lu]Lu-DPI-4452 for Human CAIV, CAXII, and CAXIV

CA protein	K_D_ (nM)	Dissociation half-life (min)
CAIV	>2,000	Not determined
CAXII	>2,000	Not determined
CAXIV	>2,000	Not determined

Affinities and dissociation kinetics were measured by surface plasmon resonance assay.

### DPI-4452 Is Rapidly Eliminated and Well Tolerated in Mice

Single intravenous DPI-4452 injections of 0.7 and 5.5 mg/kg in male CD-1 mice were well tolerated. Pharmacokinetic analysis revealed maximum plasma DPI-4452 concentrations (5 min after dose) of 1,380 and 9,270 ng/mL, respectively, and short elimination half-lives (0.28 and 0.47 h, respectively) (Table 4).

**TABLE 4.** Plasma Pharmacokinetics of DPI-4452 in Mice and Dogs

Animal	Dose (mg/kg)	AUC_inf_ (h·ng/mL)	C_5 min_ (ng/mL)	t_last_ (h)	Clearance (mL/min/kg)	V_ss_ (L/kg)	Half-life (h)
Mouse	0.7	424	1,380	2	27.5	0.41	0.28
	5.5	2,820	9,270	4	32.5	0.46	0.47
Beagle dog	0.1	133	377	2	12.7	0.26	0.38
	0.8	1,760	4,430	4	7.6	0.19	0.48

AUC_inf_ = area under plasma concentration–time curve extrapolated to infinity; C_5 min_ = measured concentration at 5 min after injection; t_last_ = time to last measurable concentration; V_ss_ = volume of distribution at steady state.

### DPI-4452 Shows Selective and High Tumor Uptake in Xenograft Mouse Models

**FIGURE 1.**
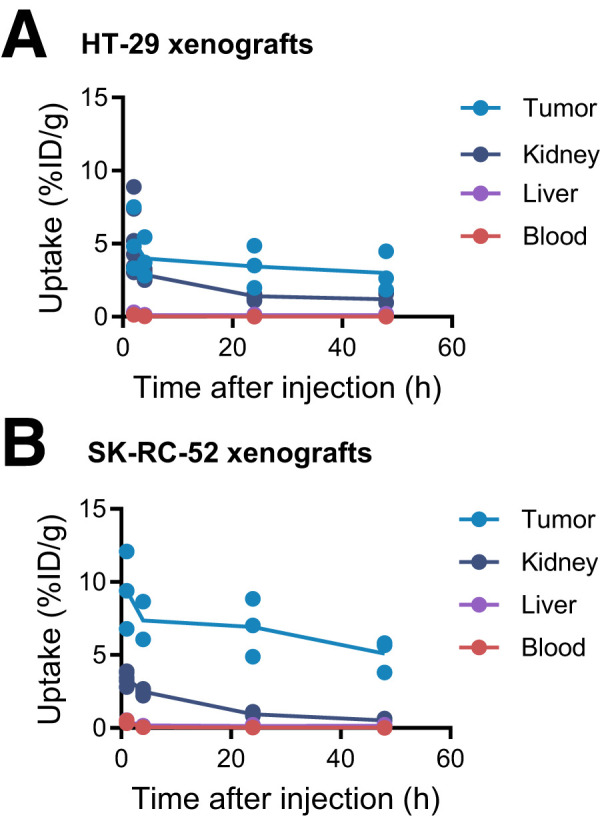
[^111^In]In-DPI-4452 tissue pharmacokinetics in mice bearing HT-29 and SK-RC-52 xenografts. Mean uptake (radioactivity concentration as percentage injected dose per gram of tissue [%ID/g]) over time in key organs after injection of 30 MBq of [^111^In]In-DPI-4452 into mice with human CRC HT-29 (A) and human ccRCC SK-RC-52 (B) subcutaneous xenografts. Shown are measurements for individual mice. Line represents mean. *n* = 3.

Radioactivity uptake was monitored up to 48 h after intravenous injection of 30 MBq of [^111^In]In-DPI-4452 in mice with subcutaneous HT-29 CRC and SK-RC-52 ccRCC xenografts using SPECT/CT imaging (Fig. 1; Supplemental Fig. 7). Maximal tumor uptake was seen at the first time point (1 and 2 h for SK-RC-52 and HT-29 xenografts, respectively). Except for the earliest time point for HT-29 xenografts, tumor-to-kidney and tumor-to-liver ratios of greater than 1 were observed for both models throughout (Supplemental Tables 2 and 3).

In both xenograft models, strong and uniform epithelial CAIX expression was observed by immunohistochemistry (Supplemental Fig. 8).

**FIGURE 2.**
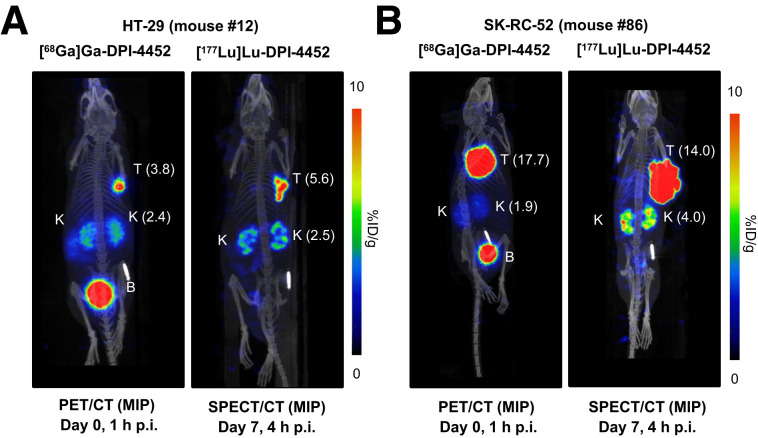
[^68^Ga]Ga-DPI-4452 and [^177^Lu]Lu-DPI-4452 biodistribution in mice with HT-29 and SK-RC-52 xenografts. Shown are maximum-intensity projection (MIP) images of representative mice bearing subcutaneous xenografts of HT-29 (A) or SK-RC-52 (B) tumors. Each mouse was imaged by PET/CT at 1 h after injection (p.i.) with 9 MBq of [^68^Ga]Ga-DPI-4452 per animal (left) and, 7 d later, by SPECT/CT at 4 h after injection with 33 MBq of [^177^Lu]Lu-DPI-4452 per animal (right). Scale bar represents radioactivity concentration as percentage injected dose per gram of tissue (%ID/g) (values in parentheses). B = bladder; K = kidney; T = tumor.

Theranostic pairs must localize to the same tissues at comparable levels to maximize their diagnostic utility and ability to predict potential treatment benefit. To address this issue, mice with HT-29 and SK-RC-52 xenografts were injected with [^68^Ga]Ga-DPI-4452 and imaged using PET/CT 1 h later. One wk later, the same mice received [^177^Lu]Lu-DPI-4452 and were imaged using SPECT/CT 4 h later. Comparably high tumor uptake and low background organ uptake were seen (Fig. 2).

### [^177^Lu]Lu-DPI-4452 Reduces Xenograft Tumor Burden

**FIGURE 3.**
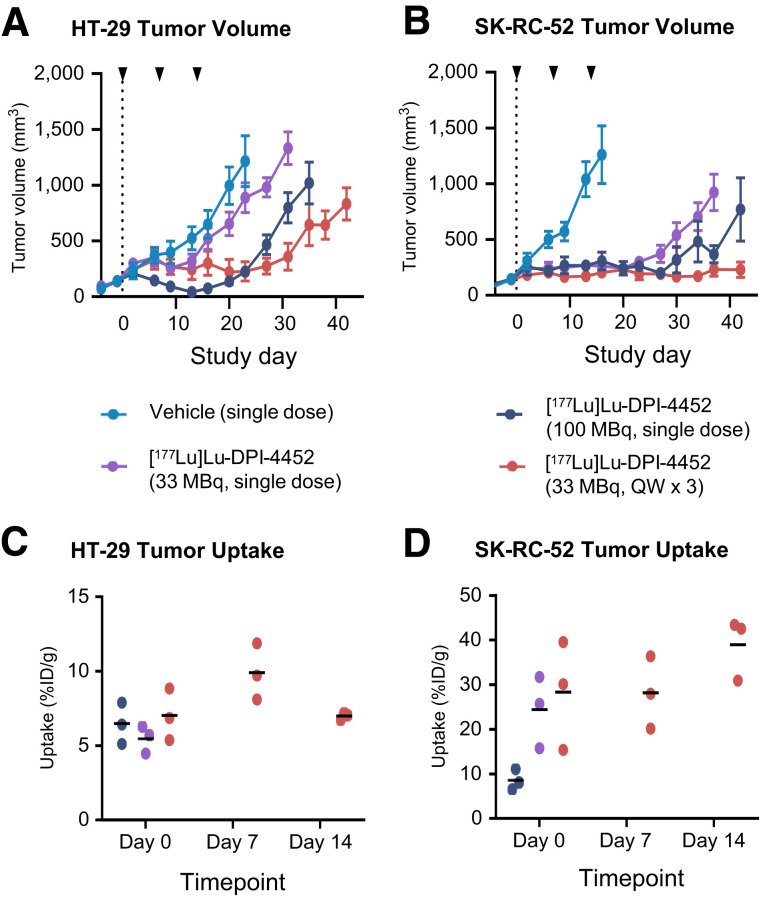
Uptake and treatment effect of [^177^Lu]Lu-DPI-4452 in subcutaneous HT-29 and SK-RC-52 human xenograft mouse models. (A and B) Growth of HT-29 (A) and SK-RC-52 (B) xenografts treated with single doses of vehicle, 100 or 33 MBq of [^177^Lu]Lu-DPI-4452, or 3 once-weekly (QW × 3) 33 MBq doses of [^177^Lu]Lu-DPI-4452. Arrowheads indicate days of treatment (*n* = 10 per group). (C and D) Tumor uptake (percentage injected dose per gram of tissue, %ID/g) for HT-29 (C) and SK-RC-52 (D) xenografts at 4 h after injection (*n* = 3 per group). Shown are mean (bar) and individual values.

The antitumor effects of [^177^Lu]Lu-DPI-4452 were assessed in HT-29 and SK-RC-52 xenografts. Tumor-bearing mice were randomized into 4 groups and injected with single doses of vehicle, 100 or 33 MBq of [^177^Lu]Lu-DPI-4452, or 3 once-weekly 33-MBq doses of [^177^Lu]Lu-DPI-4452. Treatment was well tolerated, including no significant changes in kidney function biomarkers (Supplemental Figs. 9–11). All treatment groups, except HT-29 xenograft-bearing mice receiving a single dose of 33 MBq, had significantly reduced tumor volumes compared with vehicle controls by day 16 (HT-29) or day 13 (SK-RC-52) (Fig. 3; Supplemental Figs. 12 and 13; Supplemental Table 4). Both 100-MBq and 3 once-weekly 33-MBq doses produced maximal tumor growth inhibition at day 23 for HT-29 xenografts and day 36 for SK-RC-52 xenografts, although 3 once-weekly 33-MBq doses produced a more sustained effect than a single 100-MBq dose in both models. No significant difference in tumor size for SK-RC-52 xenografts was observed at day 36 after treatment with single 100- or 33-MBq doses of [^177^Lu]Lu-DPI-4452 (Supplemental Table 4). These findings were consistent with the improved survival seen in treated mice compared with control mice (Supplemental Fig. 14; Supplemental Table 5). SPECT/CT analysis of 3 mice with xenografts per treatment arm revealed high tumor uptake in both models that was not modulated by repeated [^177^Lu]Lu-DPI-4452 treatments (Fig. 3). Similar radioactivity was found in SK-RC-52 tumors for all doses, suggesting that uptake was already maximal at the lowest dose of 33 MBq (Supplemental Fig. 15). These findings demonstrated a strong antitumor effect of [^177^Lu]Lu-DPI-4452 on CAIX-expressing tumors and suggested dose fractionation may be beneficial.

### DPI-4452 Binds cCAIX with Affinity Similar to hCAIX

Of cCAIX, rat CAIX, and mCAIX homologs, hCAIX shares the greatest amino acid homology with cCAIX. In cell-binding assays, hCAIX- and cCAIX-expressing Chinese hamster ovary cells displayed similar affinities for [^111^In]In-DPI-4452 (K_D_, 0.3 nM); these affinities were significantly higher than those for mCAIX-expressing Chinese hamster ovary cells (K_D_, 63 nM) (Table 5). When surface plasmon resonance was used, cCAIX bound [^nat^Lu]Lu-DPI-4452 with a subnanomolar K_D_ and a 69-min dissociation half-life (Supplemental Table 6), revealing an affinity comparable to that of hCAIX. Overall, the cross-reactivity of DPI-4452 and its complexes with cCAIX and hCAIX showed that the dog is a suitable species for the preclinical assessment of DPI-4452.

**TABLE 5.** Affinities of [^111^In]In-DPI-4452 for Cells Expressing hCAIX, cCAIX, and mCAIX

Species of CAIX	K_D_ (nM)
Human	0.3
Dog	0.3
Mouse	63

Cellular binding was assessed using γ-counter.

### DPI-4452 Is Rapidly Eliminated in Dogs

In a pharmacokinetic study after a single intravenous dose of 0.1 mg/kg in beagle dogs, the plasma half-life of DPI-4452 was 0.38 h, denoting rapid elimination of the peptide from the systemic circulation (Table 4). Plasma exposure (area under the concentration–time curve) increased more than dose-proportionally from 0.025 to 0.8 mg/kg in a dose-finding experiment (Supplemental Table 7), as well as in a good laboratory practice–compliant single-dose study (Table 6).

**TABLE 6.** Mean Plasma Pharmacokinetics of DPI-4452 After Single Injection of DPI-4452 in Beagle Dogs

Dose (mg/kg)	Sex	C_5 min_ (ng/mL)*	t_last_ (h)^†^	AUC_inf_ (h·ng/mL)*	AUC_inf_/dose (h·ng/mL)/(μg/kg)*
0.016	Female	37.7 (6.83)	1 (1–1)	12.4 (2.13)	0.774 (0.133)
	Male	43.5 (2.75)	1 (1–1)	14 (1.29)	0.873 (0.0806)
0.08	Female	346 (34.8)	3 (2–3)	144 (15.3)	1.8 (0.192)
	Male	332 (14.7)	2 (2–3)	132 (5.49)	1.65 (0.0687)
0.4	Female	1,760 (180)	3 (3–3)	771 (40.6)	1.93 (0.101)
	Male	1,840 (257)	3 (3–3)	854 (120)	2.13 (0.3)

* Reported as mean, with SD in parentheses.

† Reported as median, with range (minimum to maximum) in parentheses.

C_5 min_ = measured concentration at 5 min after injection; t_last_ = time to last measurable concentration; AUC_inf_ = area under plasma concentration–time curve extrapolated to infinity.

### Biodistribution of DPI-4452 in Beagle Dogs

**FIGURE 4.**
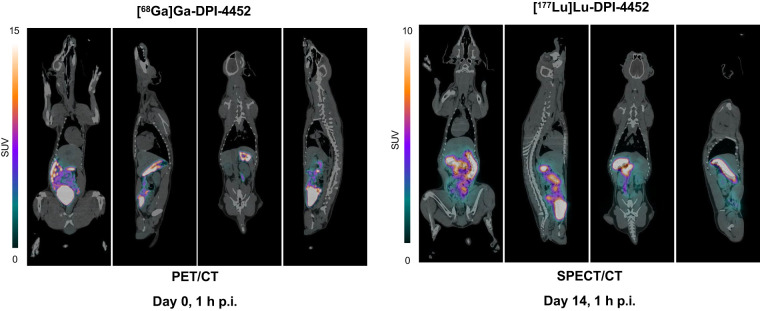
Comparison of [^68^Ga]Ga-DPI-4452 and [^177^Lu]Lu-DPI-4452 biodistributions in healthy dogs. PET/CT images taken at 1 h after injection (p.i.) of [^68^Ga]Ga-DPI-4452 at 200 MBq (left) and SPECT/CT images taken at 1 h after injection of [^177^Lu]Lu-DPI-4452 at 1000 MBq (right) for one female beagle dog (with 2-wk interval between 2 injections). Scale bars represent SUV.

**FIGURE 5.**
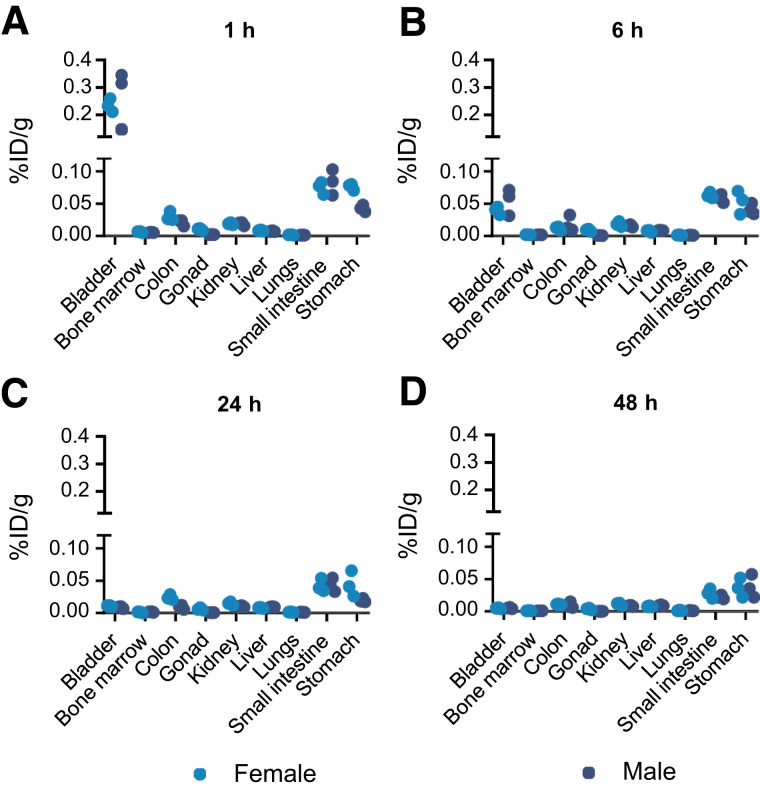
[^177^Lu]Lu-DPI-4452 tissue distribution in healthy dogs. [^177^Lu]Lu-DPI-4452 organ uptake (radioactivity concentration) for female and male beagle dogs at 1 h (A), 6 h (B), 24 h (C), and 48 h (D) after injection of 1000 MBq. Uptake (radioactivity concentration) is presented as percentage injected dose per gram of tissue (%ID/g). *n* = 3.

DPI-4452 biodistribution was evaluated in male and female beagle dogs. Each animal received a single dose of 200 MBq of [^68^Ga]Ga-DPI-4452 followed, 14 d later, by a single dose of 1,000 MBq of [^177^Lu]Lu-DPI-4452. Organ uptake of radioactivity over time was determined using PET/CT imaging and SPECT/CT imaging, respectively. At early time points, significant uptake occurred in the bladder, likely due to renal elimination. The highest sustained uptake was seen in the stomach and small intestine (Fig. 4), consistent with CAIX expression in these organs (1,2,5,18). No or low uptake was seen in the rest of the body, namely, the kidneys, bone marrow, blood, heart wall, and skin (Fig. 5; Supplemental Fig. 16).

**FIGURE 6.**
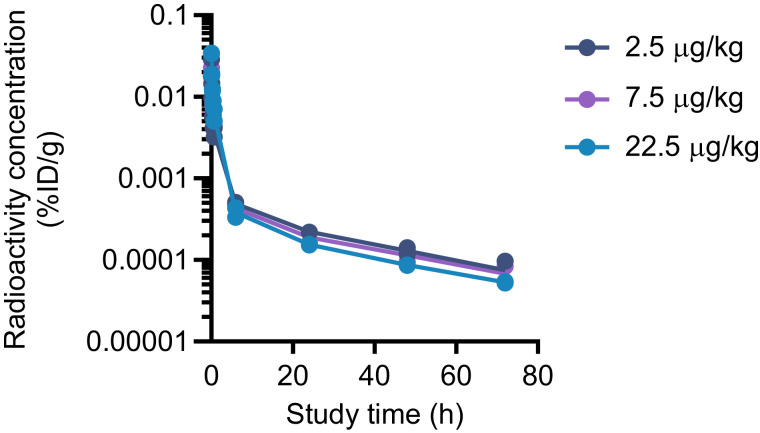
[^111^In]In-DPI-4452 plasma pharmacokinetics in healthy dogs. Shown is mean plasma radioactivity concentration (percentage injected dose per gram [%ID/g]) after injection of [^111^In]In-DPI-4452 at 25 MBq/kg with variable specific activities. Shown are individual measurements. Line represents mean. *n* = 2.

Beagle dogs were injected with a single [^111^In]In-DPI-4452 dose of 25 MBq/kg with a specific activity of 1.11, 3.33, or 9.99 MBq/μg, correlating to a ligand mass dose of 22.5, 7.5, or 2.5 μg/kg, respectively. No differences in the plasma profile of total radioactivity (Fig. 6) or organ uptake were seen (Supplemental Fig. 17), suggesting that the peptide mass dose did not affect uptake in healthy tissues in the tested dose range.

### Dosimetry of [^68^Ga]Ga-DPI-4452 and [^177^Lu]Lu-DPI-4452 in Dogs and Extrapolation to Humans

Given the assumption that CAIX organ expression is similar in dogs and humans (18), the biodistribution data for [^68^Ga]Ga-DPI-4452 were extrapolated, and the radiation doses absorbed in human organs per administered [^68^Ga]Ga dose were estimated by dosimetry (Table 7) (19). This approach yielded a predicted effective dose of 3.60E−02 mSv/MBq for [^68^Ga]Ga-DPI-4452—comparable to those of other ^68^Ga-labeled PET tracers (20).

**TABLE 7.** Calculated Absorbed Doses to Organs of [^68^Ga]Ga-DPI-4452 and [^177^Lu]Lu-DPI-4452 for Human ICRP-89 Phantoms

	[^68^Ga]Ga-DPI-4452 (Gy/GBq)		[^177^Lu]Lu-DPI-4452 (Gy/GBq)	
Target organ	Female	Male	Female	Male
Adrenals	1.52E−02	1.20E−02	1.56E−01	1.15E−01
Brain	1.09E−02	8.93E−03	1.41E−01	1.00E−01
Breasts	1.07E−02		1.39E−01	
Esophagus	1.17E−02	1.00E−02	1.43E−01	1.06E−01
Eyes	1.09E−02	8.95E−03	1.41E−01	1.00E−01
Gallbladder wall	1.80E−02	1.38E−02	2.89E−01	2.62E−01
Left colon	2.41E−02	1.94E−02	3.66E−01	2.73E−01
Small intestine	8.85E−02	6.62E−02	6.95E−01	5.31E−01
Stomach wall	7.19E−02	3.51E−02	8.24E−01	7.14E−01
Right colon	2.28E−02	1.85E−02	3.66E−01	2.72E−01
Rectum	3.89E−02	2.58E−02	1.50E−01	1.07E−01
Heart wall	1.77E−02	1.49E−02	1.47E−01	1.07E−01
Kidneys	2.16E−02	1.49E−02	4.38E−01	3.10E−01
Liver	1.18E−02	8.60E−03	2.90E−01	3.12E−01
Lungs	6.26E−03	4.48E−03	6.07E−02	4.63E−02
Ovaries/testes	2.08E−02	7.56E−03	1.71E−01	1.89E−02
Pancreas	1.64E−02	1.32E−02	1.57E−01	1.16E−01
Salivary glands	1.13E−02	9.65E−03	1.42E−01	1.02E−01
Red marrow	1.37E−02	1.01E−02	1.34E−01	9.88E−02
Osteogenic cells	9.98E−03	8.75E−03	1.36E−01	1.26E−01
Spleen	1.50E−02	1.09E−02	1.53E−01	1.08E−01
Thymus	1.19E−02	9.66E−03	1.43E−01	1.02E−01
Thyroid	1.12E−02	9.56E−03	1.41E−01	1.02E−01
Urinary bladder wall	5.33E−01	4.73E−01	4.76E−01	3.56E−01
Uterus/prostate	3.23E−02	2.31E−02	1.52E−01	1.06E−01
Total body	1.71E−02	1.38E−02	1.68E−01	1.23E−01

Radiation doses absorbed in human organs after [^177^Lu]Lu-DPI-4452 administration were similarly extrapolated (Table 7). High values were observed, irrespective of sex, in the stomach wall and small intestine (0.824 and 0.695 Gy/GBq, respectively, in females); however, relatively low values were obtained in the other organs, such as the kidneys and red marrow (0.438 and 0.134 Gy/GBq, respectively, in females). To our knowledge, radiation dose limits for human organs have not been established for targeted radiotherapy. According to the estimated radiation doses absorbed in human organs, none of the limits set for external-beam radiation therapy (21) should be exceeded after a dose of up to 25.9 GBq of [^177^Lu]Lu-DPI-4452.

## DISCUSSION

As a direct transcriptional target of hypoxia-inducible factor 1α, CAIX is overexpressed in several hard-to-treat tumors, including ccRCC, CRC, breast cancer, and PDAC (2,9–11), making it a promising therapeutic target. However, to date, the transition of CAIX-targeting therapies to clinical settings has been poor (7,13). Several small-molecule inhibitors of CAs, including CAIX, have been developed (7,13). The most promising of these, the sulfonamide derivative SLC-0111, was well tolerated in a phase 1 study of advanced solid tumors, but no objective responses were observed (22). Modest responses were also reported in clinical trials with the anti-CAIX monoclonal antibody G250 (13), but no improvement in survival compared with placebo was observed in a phase 3 adjuvant trial in patients with high-risk ccRCC (23). Conjugation of G250 to the β-emitter [^177^Lu]Lu resulted in improved preclinical activity (13,24,25); in 2 clinical trials of metastatic ccRCC, disease stabilization was reported for 9 of 14 and 17 of 23 patients (26,27), despite dose-limiting myelotoxicity, a common side effect of radiolabeled antibodies (28). These data suggest that although antibodies and inhibitors targeting CAIX have not, to date, shown clinically relevant antitumor efficacy, they can be used to deliver radioactivity to tumors. With CAIX expression in healthy tissues being largely restricted to the small intestine and stomach (1,2,5,18), peptide-based therapeutic agents targeting CAIX, such as [^177^Lu]Lu-DPI-4452, provide the potential to target hypoxic or von Hippel-Lindau tumor suppressor–mutated tumors while featuring a favorable biodistribution (7,13). Moreover, peptide receptor radionuclide therapy agents are usually nonimmunogenic and display favorable pharmacokinetics, characterized by high target tissue concentration and rapid excretion from the body, which is expected to limit adverse effects from prolonged exposure to circulating radioactivity (29).

Previously, CAIX-targeting peptides showed poor tumor uptake in preclinical studies (7). In the present study, DPI-4452 showed excellent tumor accumulation and lower uptake in healthy tissues. Also, [^177^Lu]Lu-DPI-4452 showed antitumor efficacy in CRC and ccRCC xenografts. Moreover, the similar responses seen for 3 33-MBq doses versus a single 100-MBq dose suggest that dose fractionation could be a viable option (Fig. 3) for the reduction of potential dose-limiting organ toxicities (30).

Because of the lack of cross-reactivity of DPI-4452 with mCAIX, mouse models provide limited relevance for investigating radiotoxicity and ligand toxicity in organs naturally expressing CAIX, whereas the similar binding of DPI-4452 to hCAIX and cCAIX suggests that the dog is a suitable species for the preclinical safety assessment of this agent.

A single administration of DPI-4452 at up to 0.4 mg/kg was well tolerated in a good laboratory practice toxicity study in beagle dogs (31). Accumulation was predominantly seen in CAIX-expressing tissues, such as the stomach and small intestine (18); in addition, transient distribution was seen in the urinary bladder, due to renal elimination. Importantly, low or no uptake was observed in other organs, including the kidneys and bone marrow. Coupled with high tumor-specific uptake in murine xenografts, this finding means that [^68^Ga]Ga-DPI-4452 could serve as a PET-based tool for selecting patients for treatment with [^177^Lu]Lu-DPI-4452.

Extrapolating [^177^Lu]Lu-DPI-4452 dog dosimetry to humans suggested the small intestine and stomach to be potential dose-limiting organs, considering the conservative limits set for external-beam radiotherapy (21). Although the dog organs with the highest uptake are those associated with high CAIX expression in humans, differences in CAIX expression between these species remain uncertain. Therefore, the first biodistribution data from humans (e.g., with [^68^Ga]Ga-DPI-4452) will provide useful insights. Accordingly, the present work provides a strong foundation for the initiation of human studies with this theranostic pair.

## CONCLUSION

This is the first report of preclinical biodistribution and pharmacologic proof-of-concept studies involving DPI-4452. [^68^Ga]Ga-DPI-4452 and [^177^Lu]Lu-DPI-4452 showed tumor-specific uptake, high tolerability, and rapid systemic elimination. [^177^Lu]Lu-DPI-4452 also demonstrated strong antitumor efficacy in 2 murine xenograft models. [^68^Ga]Ga-DPI-4452 and [^177^Lu]Lu-DPI-4452 are a promising theranostic pair for targeting CAIX-expressing tumors. A clinical phase 1/2 study is currently underway to assess the safety, tolerability, and imaging characteristics of [^68^Ga]Ga-DPI-4452 and the efficacy of [^177^Lu]Lu-DPI-4452 in patients diagnosed with CRC, PDAC, or ccRCC and having unresectable locally advanced or metastatic solid tumors (ClinicalTrials.gov identifier: NCT05706129).

## DISCLOSURE

This work received financial support from Debiopharm International SA. Franck Brichory, Frédéric Massière, Norbert Wiedemann, and Antoine Attinger are current employees and Inês Borrego was a former employee of Debiopharm International SA. Aileen Hoehne, Frank Osterkamp, Matthias Paschke, Dirk Zboralski, Anne Schumann, and Anne Bredenbeck are employees of 3B Pharmaceuticals GmbH. No other potential conflict of interest relevant to this article was reported.

## Notes

KEY POINTS **QUESTION:** Does DPI-4452 have theranostic potential for targeting CAIX-expressing tumors and is it well tolerated in relevant animal models? **PERTINENT** **FINDINGS:** DPI-4452 selectively bound CAIX, and the theranostic pair [^68^Ga]Ga-DPI-4452 and [^177^Lu]Lu-DPI-4452 showed selective tumor uptake and, for the latter, strong tumor growth inhibition in 2 murine xenograft models of CAIX-expressing tumors. Radioactivity uptake in dogs was observed in CAIX-expressing organs (small intestine and stomach); however, uptake was low in all other organs, including the kidneys and bone marrow, with rapid elimination from systemic circulation (half-life of less than 1 h). **IMPLICATIONS** **FOR PATIENT CARE:** As a direct transcriptional target of hypoxia-inducible factor 1α, CAIX is overexpressed in ccRCC and hypoxic tumors, such as CRC and PDAC; the theranostic pair [^68^Ga]Ga-DPI-4452 and [^177^Lu]Lu-DPI-4452 provides a novel selection strategy and treatment modality for targeting CAIX-expressing tumors.
